# Medical Professionalism Across Cultures: A Literature Review

**DOI:** 10.15694/mep.2019.000191.1

**Published:** 2019-10-21

**Authors:** Laith Yasin, Gerald R. Stapleton, L. J. Sandlow

**Affiliations:** 1 University of Illinois at Chicago

**Keywords:** Medical Professionalism, Ethics, Cultures

## Abstract

This article was migrated. The article was marked as recommended.

The review aims to identify the cultural perspectives of medical professionalism by identifying relevant literature from the Middle East, East/South Asia, and the Western world that discuss definitions. A literature search was conducted using the “Summon” search engine, and 200 articles sorted by relevancy were manually reviewed. Based on the surveys and documents gathered from each of the regions, the definitions seem to be fairly consistent in their recognition of characteristics important to the concept of medical professionalism. These include several characteristics, with some of the most common being personal character, respect for patient autonomy, responsibility, and social obligations; the main difference lies in emphasis with the West focusing on societal issues and patient rights, the Middle East focusing on morality and personal character, and East Asia focusing on respect, responsibility, and other duties. These differences are reviewed and the cultural sources are further expanded upon.

## Introduction

The development of a common definition of medical professionalism has been elusive for centuries. Definitions have continuously evolved as a result of changing philosophies regarding ethics. Most evidently, the Middle East, East/South Asia, and the Western world have taken different approaches to developing an overall definition of medical professionalism. Historical evidence of the definitions includes oaths and codes. More recently contributions to the definition have been assisted by surveys, scholarly reports, and syntheses of viewpoints from the ABIM, CanMEDS, and other organizations. In reviewing this evidence, we attempt to identify key documents that have impacted the interpretation of medical professionalism in various cultures through history.

## Methods

Asearch of the literature using the “Summon” search engine was utilized to assure that the full text of each article in the database was indexed so that all articles that included references to the search terms would be included and not just those with such terms in their titles or keyword indices. A search of English language articles appearing over the last 10 years with full text available online appearing in scholarly journals and books was performed using the keywords “medical ethics” and “codes” and “history”. This initial search yielded 56,271 books and articles including many that were focused on nursing and pharmacy. The authors narrowed the focus to medical practice by a second search excluding the keywords “Nursing”, “Nurses”, and “Pharmacy”. The second search yielded 25,910 articles. These articles were sorted by relevance using the Summon search metrics and the 200 articles and books that were determined to be most relevant were manually reviewed by the lead author with the intention of identifying references to key sources that were regarded as influential in establishing the meaning and interpretation of medical professionalism through history and across cultures. No new documents were identified in the final quarter of the selected search results leading us to conclude that the sample selected was satisfactory to identify the key sources of influence through history and across various cultures.

## Findings and Discussion

### Western Definitions

The Western definition of professionalism has been extensively documented in the literature and source documents show clear signs of evolution as a result of changing cultures and ideas. In Western cultures, the Hippocratic Oath has regularly been recognized as a key milestone in the development of medical professionalism, laying a groundwork for the explicit description of physician responsibilities. Charles Bryan’s analysis ( Bryan, 2011) of the development of medical professionalism references four pivotal events, the Hippocratic Oath being the first to occur, followed by the conception of medical ethics, the Flexner Report, and the commoditization of medicine. Bryan emphasizes the importance of developing a definition of medical professionalism in each generation, in order to defend medicine’s status as a “learned profession” ( Bryan, 2011). However, in a study conducted by Fabrice Jotterand ( Jotterand, 2005), the relevance of the Hippocratic oath is questioned. Jotterand notes that many view the Hippocratic oath’s influence to be diminished in contemporary medicine, due largely to outdated views on morality ( Jotterand, 2005). On the other hand, defense of the oath’s influence largely refers to its symbolic status as a foundation of medical ethics. Further, the author notes various arguments that rest on the implied social and political obligations of the oath, as well as its implied leaning towards a universal healthcare system ( Jotterand, 2005). Overall, Jotterand asserts the importance of developing an overall philosophy of medicine, in order to substantiate the principles described in the oath ( Jotterand, 2005).

In an article assessing the development of professionalism through various oaths and codes, Garrick Applebee cites the Hippocratic oath as the first major event in the progression of medical professionalism ( Applebee, 2016). He further notes that the oath was generally seen as a personal oath prior to the Middle Ages, when it became a standard for professionalism in medical schools ( Applebee, 2016). Similar to Bryan’s article, Percival’s “Medical Ethics” and the Flexner Report were also cited by Applebee as key milestones, with the addition of other codes, like Sun Simiao’s oath, the AMA code of ethics, and the ABIM physician charter (Applebee, 2016). The documents show a progression in the specific attributes, moving from duties of the physician to concepts like patient rights and social justice ( Applebee, 2016). In an article ( Cohen 2006) aiming to develop a definition of professionalism, Cohen mentions the effects of the Hippocratic Oath, Maimonides, and William Osler to show the ubiquity of certain aspects of professionalism; namely, the “primacy of patient interest”. This generally conflicts with the idea that the Hippocratic oath has been mostly phased out, as it shows the clear retention of major concepts over the course of history.

The comparisons between the Hippocratic oath and modern professionalism are further elaborated on in an article by Friedrich Heubel ( Heubel, 2014), in which the concepts of patient welfare and self-determination are determined to be consistent between the oath and the ABIM charter on professionalism. However, the main difference stems from the concept of social justice detailed in the charter, calling for fairness in the distribution of health resources. As noted previously, some defenders of the oath claim that universal health care is implied within the oath. The explicit call for social justice in the ABIM charter represents a notable difference, however, regardless of the deeper interpretation of the Hippocratic oath.

More recently, the AMA Code of Ethics has been a key example of the contemporary view of medical professionalism in the West, with several revisions in the past two centuries. As Baker and Emanuel describe in an article ( Baker and Emanuel, 2000) analyzing the AMA Code’s relevance, the code put patients’ interests above the professional, following the tradition set by the Hippocratic Oath. The authors also emphasize the most common themes, primarily the focus on the physician-patient relationship and the obligation to advise the public in all matters ( Baker and Emanuel, 2000). Notably, in an article discussing medical malpractice and medical professionalism by Klaas *et al.* ( Klaas *et al.,* 2014), a substantial change was observed in the 1980 revision, with the change from “not speaking ill of a physician’s competence” to an obligation to “report physicians deficient in character or competence”. This shows a general trend toward less protection for physicians and an increasing emphasis on giving patients the best care possible.

Many have accepted the ABIM physician charter as a modern definition of medical professionalism and the charter’s attributes are often used as a starting point for a Western definition. The charter outlines three fundamental principles, Primacy of Patient Welfare, Patient Autonomy, and Social Justice, along with several professional responsibilities.

Not all have viewed the ABIM charter uncritically. In a critique of the physician charter ( DeAngelis, 2015), DeAngelis cites the difficulty in pinpointing a definition that includes all the attributes one would expect of a physician. In particular, the article emphasizes the lack of the term “compassion” in the physician charter, despite its prevalence in definitions around the world, as noted by the studies in East Asia and the Middle East reviewed below ( DeAngelis, 2015). DeAngelis notes the potential for physicians to place the welfare of the institutions employing them over the care of their patients ( DeAngelis, 2015). Swick *et al.* describe the primary problem with the physician charter to be its basis as a contract ( Swick *et al.,* 2006). The authors explain that the reference to the physician-patient relationship as a contract connotes a formal agreement based on distrust, further supported by the restrictive tone of the charter ( Swick *et al.,* 2006). With an attitude of distrust between physicians and patients, physicians may provide care more rigidly, focusing on their legal obligations rather than on optimizing care for the patient. Further, the authors describe the value of medical oaths to the profession, as providing a basis for morality and medical professionalism which in turn acts to prevent the commercialization of medicine ( Swick *et al.,* 2006). Clearly, Western professionalism is centered around the maintenance of the profession, focusing on physician autonomy and the physician-patient relationship in an effort to limit the commercialization of medicine and the risk of a more business-oriented distribution of healthcare.

The CanMEDS definition of professionalism is another highly regarded representative of the Western ideals. The differences between this definition and the ABIM Charter is described in Reid’s article ( Reid, 2011) regarding medicine as a social contract. Generally, the physician charter lays out the obligations of the physician to society. In contrast, the CanMEDS definition represents more of an exchange between physicians and society, describing the obligations and expectations of both parties ( Reid, 2011). While this does more in terms of giving physicians more autonomy, preventing the potential for rigid, strict medical practice, it still contain the same problems associated with trust detailed in Swick’s previously mentioned article. This exchange detailed in the CanMEDS definition was notably derived from Richard and Sylvia Cruess’ definition of professional roles, which CanMEDS adopted in response to calls by the Canadian public for limitations on physicians in the 1980’s ( Reid, 2011).

In an effort to gain a different perspective on medical professionalism, Chandratilake *et al.* conducted a survey ( Chandratilake *et al.,* 2010) of a representative sample of the American public and found that, as expected, the public placed the most importance on concepts regarding patient rights, such as patient autonomy and confidentiality. The least emphasis was placed on physician interactions with colleagues and teamwork related attributes ( Chandratilake *et al.,* 2010). It is noteworthy, however, that the public listed all kinds of attributes, which the author separates into three categories: clinicianship, workmanship, and citizenship ( Chandratilake *et al.,* 2010). Thus, though the emphasis on particular attributes varies between the public and physicians, the general public still recognizes the obligations of physicians to patients, colleagues, and society. The modern tone for professionalism places much more emphasis on physician relationships beyond the typical doctor-patient interactions, with social obligations becoming prominent in most definitions of Western professionalism.

Other sources for prominent definitions are reviewed in an article by Hilton and Southgate ( Hilton and Southgate, 2007), showing the strong influences of attributes laid out by Swick, Calman, and Epstein, each of whom push for a normative definition of medical professionalism. These approaches generally focus on the attributes of the physician, as opposed to a contract-based structure. These attributes include several that align with public opinion, such as respect for patients and responsibility to the patient and society. They also comprise the personal aspects of professionalism, detailing the need for physicians to have focus and attention, actively managing their mental approach to patient care ( Hilton and Southgate, 2007).

### Middle Eastern Definitions

While many common definitions of medical professionalism are based on Western values, having originated mainly in Europe, the Middle East generally has taken an Islamic perspective on the basic values outlining the concept ( Kasule, 2013). According to Kasule, the Islamic definitions emphasize seven basic values, consisting of: faith, consciousness, best character, excellent performance, striving toward perfection, responsibility, and self-accountability, as opposed to the three primary principles of professionalism and various professional responsibilities outlined in the ABIM physician charter ( Kasule, 2013). As Kasule states, the primary difference between the two proposed definitions lies in the Islamic emphasis on morality and personal character, specifically a humble attitude and sincerity in human interactions ( Kasule, 2013). However, definitions of professionalism in the Middle East rely heavily on the religious background, as religion plays a large role in perspectives of morality and ethics.

Al-Eraky *et al.*, in a survey of medical students from universities in Egypt and Saudi Arabia ( Al-Eraky *et al.,* 2013), found that students placed the most importance on concepts generally related to “respect for others”, which included both patients and the healthcare team. Meanwhile, the lowest overall scores of importance were generally found in the “honor/integrity” related concepts, which centered primarily around disclosure of information relevant to the patient ( Al-Eraky *et al.,* 2013). While the Arabian context was the focus of the survey, the characteristics surveyed were derived mainly from the ABIM. Nonetheless, the student emphasis on respect in relationships seems to fall in line with the Islamic definition’s emphasis on personal character and human interactions Kasule outlines ( Al-Eraky *et al.,* 2013). In a survey of advanced Kuwait medical students ( Al-Abdulrazzaq *et al.,* 2014), Al-Abdulrazzaq *et al.* found that Kuwait students most commonly defined aspects of professionalism related to punctuality, respect, and attire, with the least common attributes being skill, wisdom, and considerate behavior. The survey analysis used CanMEDS competencies as a framework to assess the student definitions, which showed students placing most emphasis on ethical behavior, while lacking in areas related to physician health and social involvement regarding regulation ( Al-Abdulrazzaq *et al.,* 2014). The authors state the prevalence of “respect” was seen throughout similar studies, as described previously in Al-Eraky and Kasule’s results regarding Islamic and Arabian definitions of medical professionalism, depicting the historical influence of Muslim medicine from the Islamic-Arabic Golden Age.

Al-Eraky *et al*. formulated an Arabian definition to eight attributes based on medical experts’ opinions from universities in Egypt, Saudi Arabia, Oman, and Sudan. These attributes were then sorted in a Four-Gates Model, with the broader categories being: dealing with self, dealing with tasks, dealing with others, and dealing with God ( Al-Eraky *et al.,* 2014). The qualities derived from the expert responses were compared to Western definitions using the ABIM Physician Charter and other scholarly reports, sharing characteristics such as respect, accountability, and excellence ( Al-Eraky *et al.,* 2014). The primary difference between the study results and the Western definitions was the fourth “gate”: dealing with god, which placed emphasis on self-accountability and self-motivation in relation to core religious values of Islam ( Al-Eraky *et al*., 2014). In modern Western texts, aspects of religion in medical professionalism have not been discussed in great detail, though, in contrast, they play a central role in Arabian definitions of medical professionalism.

Al-Eraky and Chandratilake further propose an Arabian definition of medical professionalism based on a panel of healthcare professionals from institutions in Egypt, Saudi Arabia, and United Arab Emirates ( Al-Eraky and Chandratilake, 2012). The results indicated an overall acceptance of the ABIM Physician Charter’s six domains, while further adding “autonomy of professionals” to the list ( Al-Eraky and Chandratilake, 2012). Though all domains were rated highly, honor/integrity received the highest score of importance, while excellence received the lowest ( Al-Eraky and Chandratilake, 2012). This is contrary to Al-Eraky’s survey mentioned previously, in which medical students in the same region gave the lowest importance rating to the same category. The addition of autonomy of professionals emphasizes the power given to physicians in regards to decision-making, as opposed to the western practice of giving more power to patients in that respect ( El-Eraky and Chandratilake, 2012). Overall, the modern definition of medical professionalism in the Middle East, as indicated by the surveys conducted and definitions proposed, aligns with the ABIM, with amendments in the form of religious addendums and an emphasis on personal character.

Similarly, modern Jewish Medical Ethics, as described by Jotkowitz in an article outlining the contributions of Rabbi Moshe Feinstein ( Jotkowitz, 2014), has been shaped heavily by religious principles.Themes have shifted towards more patient-centered care, with patient autonomy and informed consent being a key emphasis ( Jotkowitz, 2014). Perspectives on euthanasia, abortion, artificial reproduction, and other areas have been commonly dictated by Jewish Law, though the modern interpretations have shifted and allowed for development of new principles of medical ethics ( Jotkowitz, 2014). Thus, the impact of religious texts and ancient sources is evident in similar fashion to the Islamic approach to medical professionalism. The religious aspects are further evidenced by Maimonides Prayer, which focuses on duties of the physician, emphasizing their character and intention. The prayer also was one of the earliest to include the obligations of the physician to recognize their uncertainty and limitations, and to continuously pursue knowledge.

### East/South Asian Definitions

Much of the literature regarding East Asia’s perception of medical professionalism is similarly in the form of surveys of students and professionals. In a survey of Korean physicians conducted by Kim and Choi ( Kim and Choi, 2015), it was found that duties, such as veracity and responsibility were held in higher regard than virtues, such as altruism and honesty. The authors also state that the healthcare system in place, which is heavily government-led, has resulted in loss of autonomy for physicians, creating a more rigid practice and diminishing characteristics like altruism ( Kim and Choi, 2015). Interestingly, the author also cites the impact of Confucian thought, particularly on older physicians. Confucian thought emphasized the concept of Zi Zhong, which has been generally related to integrity, indicating the source of the higher importance rating for the concept among older physicians ( Kim and Choi, 2015). In an article assessing the historical development of medical professionalism in Korea ( Hahm and Lee, 2012), Hahm and Lee claim that Confucianism shaped early ethics, focusing on social positions and responsibilities. Ethics continued to develop, focusing primarily on physician etiquette, with the revisions of the Korean Medical Association’s Doctor’s Code of Ethics failing to instill an “ethics tradition” into the practice ( Hahm and Lee, 2012).

In a comparison of two Chinese contexts, Ho *et al.* surveyed Chinese and Taiwanese students and physicians’ opinions on a framework to define medical professionalism ( Ho *et al.,* 2014). Between the two contexts, the primary difference was the Chinese preference of “morality” as a core competency in place of “integrity” preferred by the Taiwanese ( Ho *et al.,* 2014). The authors cite the similarities of the Taiwanese and Chinese frameworks for professionalism as being rooted in Confucianism, evidenced by the prominence of morality and integrity. The survey also showed the distinction from Western professionalism, as the both Chinese cultural groups placed more emphasis on morality and personal/social roles, citing the CanMEDS competencies as an example for the lack of attention to personal factors ( Ho *et al.,* 2014). The authors also cited the economic considerations of the Chinese group as opposed to the lack of acknowledgement from the Taiwanese participants, referencing the regions respective healthcare policies as a probable explanation ( Ho *et al*., 2014). That is, Chinese opinions were observed to be clearly influenced by economic policy that was unique to mainland China, showing distinction from Taiwan.

Other surveys of Chinese and Taiwanese students and physicians further depict the East Asian perspective of medical professionalism. In a survey of first-year Chinese medical students ( Jiang *et al.,* 2010), Jiang *et al*. shows a strong emphasis on ethics when defining the attributes of professionalism. It is further noted that the attributes gathered from the student responses generate a simplified response when compared to the perspective of professionals, as expected with their limited experience ( Jiang *et al*., 2010). Similarly, in a sample of seventh year Taiwanese students, Tsai *et al*. discovered that students place the most emphasis on accountability and respect ( Tsai *et al*., 2007). Notably, however, the scores were all relatively high in terms of importance, with more emphasis on the more broad aspects of professionalism in terms of patient interactions ( Tsai *et al*., 2007). Meanwhile, in a study of thirteen occupational groups (volunteers, students, public health experts, physicians, etc.), Pan *et al*. noted different levels of value associated with aspects of professionalism, depending on their professional status ( Pan *et al*., 2013). Of all the surveyed groups, clinical competence was consistently ranked the most important attribute, with groups showing main differences in the addition of attributes like self-management, teamwork, and health promotion ( Pan *et al*., 2013).

In a survey of Chinese physicians, Nie *et al*. determined cross-cultural differences and commonalities between as compared to American attitudes on professionalism ( Nie *et al*., 2015). The survey found that Chinese physicians generally supported the principles of the ABIM Physician Charter, primarily focused on issues of consent, full disclosure, and patient autonomy ( Nie *et al*., 2015). The authors determined that Chinese culture and medical ethics are generally compatible in regards to moral expectations.

In a review of medical professionalism research published in the Chinese language, Wang *et al*. identified four main themes of research: teaching professionalism, practicing professionalism, conceptualizing professionalism, and teaching professionalism ( Wang *et al*., 2016). In their conceptualization, Chinese authors typically examine professionalism in either religious or political contexts ( Wang *et al*., 2016). Philosophies of traditional Chinese medicine are derived from Confucian or Taoist thought, and authors make the argument that their values, such as benevolent practice, self-discipline, and harmony, should make up the foundation of medical professionalism ( Wang *et al*., 2016). The Chinese Communist’s Party’s values were also analyzed, as authors aligned the core socialist values, such as freedom, justice, and rule of law, to core principles of medical professional ethics, such as autonomy, beneficence, and justice. Wang *et al*. concluded that the in terms of conceptualizing a definition, the primary concern was moving towards prioritization of patient welfare over self-interest ( Wang *et al*., 2016).

One example of a Chinese Code of Ethics was outlined in an article, where Ip analyzed the development of medical ethics in the 1990’s ( Ip, 2005). In this Code of Conduct, relationships of the physician were emphasized, giving a framework for physician conduct with patients, colleagues, and society ( Ip, 2005). Notably, the code was very general, making the measure of one’s professionalism and ethical performance more subjective. Interestingly, the Code of Conduct does not seem to correlate with the responses from the surveys of Chinese students and professionals described previously, indicating that the very general tone may have led to failed implementation or accountability of the standards outlined.

In contrast, the Indian Code of Ethics analyzed in an article by Benner has more specific standards, and a focus more on physician duties than relationships ( Benner, 2005). However, it does share the same principles of respect for patients, colleagues, teachers, and all other relationships ( Benner, 2005). Over the course of the 1900s, the principles have not changed significantly, though the wording has been adjusted to be more politically correct. Benner also notes that India generally aimed to align with international standards in order for physicians trained to practice worldwide ( Benner, 2005). Thus, their Code of Ethics closely follows the standards of the World Medical Association, which focus on the duties of the physician in general, and to their patients ( WMA, 2018).

The East Asian definition of medical professionalism shows a strong emphasis on respect, responsibility, and other duties, with less focus on the state of physicians themselves in terms of personal health. More evidently, the literature suggests slight differences in the definition of medical professionalism between different groups, with more emphasis on attributes more relevant to their given status, notably with students as opposed to practicing physicians.

In 2008 and 2009, one of us (LJS) surveyed the faculty of four medical schools regarding their definition of Professionalism. The schools included Chiba University School of Medicine in Chiba, Japan; Shanghai College of Medicine in Shanghai, China, Princess of Naradhiawas University Medical School in Thailand, and Jordan University of Science and Technology School of Medicine in Jordan. The faculties were asked to list those characteristics that they felt defined the term “Professionalism”. Of note, the Chiba Dean informed him that there was no word for Professionalism in Japanese. These surveys were performed within a workshop session and the characteristics were summarized and then discussed as well as applied in some case studies. The results of these sessions were compared to the responses of US residents in their online learning module on professionalism. In general, the faculty of the 4 medical schools in the Middle East and Asia agreed with the characteristics provided by the GME residents in the United States, which included integrity, altruism, responsibility, confidentiality, and leadership among other characteristics commonly associated with medical professionalism.

## Conclusion

Based on the surveys and documents gathered from each of the regions, the definitions seem to be fairly consistent in their recognition of characteristics important to the concept of medical professionalism. These include several characteristics, with some of the most common being personal character, respect for patient autonomy, responsibility, and social obligations. The main difference lies in emphasis, where the Western definition focuses on societal issues and patient rights, the Middle East focuses on morality and personal character, and East Asia focuses on respect, responsibility, and other duties. Some of this distinction comes from the potential separation of medical professional characteristics into what Cruess and Cruess outlined as traits of the healer, and those of the professional (Cruess, Cruess, and Steinert, 2009). While some regions may focus primarily on the professional characteristics, others may place more emphasis on the healer, leading to the absence of characteristics when determining a definition for medical professionalism.

## Take Home Messages

• Much of the difference in Medical Professionalism across cultures lies in emphasis rather than definition
• Western definition emphasizes societal issues and patient rights
• Middle East definitions emphasize morality and personal character
• East/South Asian definitions emphasize respect, responsibility, duties

## Notes On Contributors

Laith Yasin, MD Candidate 2020, is a student at the University of Illinois College of Medicine.

Gerald R. Stapleton is a member of the faculty in the Department of Medical Education at the University of Illinois at Chicago where he served as the Director for Distance Education from 1999 through 2018.

L. J. Sandlow, MD is Emeritus Professor of Medicine and Medical Education in the College of Medicine at the University of Illinois at Chicago. He was previously the Senior Associate Dean for Academic & Educational Affairs and the Head of the Department of Medical Education at the College of Medicine, UIC.
