# Supplementary material for: Medical Professionalism Across Cultures: A Literature Review
Source: MedEdPublish (2016). 2019 Oct 21;8:191. [Version 1] doi: 10.15694/mep.2019.000191.1 (PMC10712535; doi:10.15694/mep.2019.000191.1)
Supplement: Peer review history

# Reviewer report: Reviewer response for version 1

**DOI:** 10.21956/mep.19924.r30208

**Published:** 2019-11-05

**Authors:** Judy McKimm

**Affiliations:** 1 Swansea University

This review has been migrated. The reviewer awarded 3 stars out of 5 I read this paper with interest and note the comments of other reviewers also. I learned some new aspects about professionalism which probably reflects the way in which the search was carried out. Great to see a medical student included as an author as well.I think this would be improved by some summary tables, especially contrasting the different regions, there are some missing regions (Africa, South America, Australasia) and also I would think some papers regarding indigenous peoples from around the world could have been included. As noted, one of the main contrasts is between countries/regions that are more or less secular and there is a shift in many western countries towards a more secular society so the societal contract is changing between doctors and society.A useful addition to our understanding of professionalism.

Reviewer Expertise:

NA

No decision status is available

# Reviewer report: Reviewer response for version 1

**DOI:** 10.21956/mep.19924.r30207

**Published:** 2019-10-25

**Authors:** P Ravi Shankar

**Affiliations:** 1 American International Medical University

This review has been migrated. The reviewer awarded 5 stars out of 5 Thank you for the invitation to review this interesting paper. The authors have compared medical professionalism across western, Middle Eastern and East Asian cultures. The article is well written and the quality of written English is good. I am also happy to note that the first author is a medical student. One reason for the possible differences could be the diminishing role of religion in western and East Asian societies. In the west since the middle ages the role of religion is diminishing in most regions though exceptions remain. In China the Communist party has tried to reduce the role of religion in everyday life. The issue is more complex however if we look at it in terms of geographical areas. With migration to the west from other regions western thought and philosophy is also getting influenced by Asian thought processes. I am happy that Jewish philosophy is also briefly addressed in the Middle East section. The Middle East has seen a lot of migration from other regions in recent times. In the East Asian section there are other minority cultures like the Tibetan and the Islamic traditions which may have their own perspectives on professionalism. Also due to the economic growth in East Asia migration from other regions including the west is happening which can influence cultures and traditions. In the modern world due to ease of communication and transfer of thoughts and ideas various cultures are more easily able to influence one another. The article mainly addresses professionalism in terms of published research. The article is long and I believe that figures and a few tables summarizing important aspects of the discussion would be useful. This is an important paper dealing with a topic of vital importance to all educators.

Reviewer Expertise:

NA

No decision status is available

# Reviewer report: Reviewer response for version 1

**DOI:** 10.21956/mep.19924.r30212

**Published:** 2019-10-23

**Authors:** Michelle McLean

**Affiliations:** 1 Bond University

This review has been migrated. The reviewer awarded 3 stars out of 5 The primary author of this publication is a student. Congratulations for grappling with a convoluted topic. I had to concentrate while reading the body of the paper but the Conclusion is succinct and draws out the main difference (emphasis not difference). For someone starting out in the field, I think that this will be a useful summary of some of the key considerations when interpreting the reams of publications in the area. Noting the concerns of previous reviewers, I would still recommend this for those working in the field.

Reviewer Expertise:

NA

No decision status is available

# Reviewer report: Reviewer response for version 1

**DOI:** 10.21956/mep.19924.r30210

**Published:** 2019-10-23

**Authors:** Junichi Tanaka

**Affiliations:** 1 tohoku university

This review has been migrated. The reviewer awarded 4 stars out of 5 I read it very interestingly. I wanted to recommend a paper written by Dr. Nishigori about Japanese professionalism and Bushido.Acad Med. 2014 Apr;89(4):560-3. doi: 10.1097/ACM.0000000000000176.Bushido and medical professionalism in Japan.

Reviewer Expertise:

NA

No decision status is available

# Reviewer report: Reviewer response for version 1

**DOI:** 10.21956/mep.19924.r30211

**Published:** 2019-10-22

**Authors:** PATRICIA CURY

**Affiliations:** 1 FACERES

This review has been migrated. The reviewer awarded 4 stars out of 5 It is a very interesting review, many information about different cultures, but I missed african and south American point of view...

Reviewer Expertise:

NA

No decision status is available

# Reviewer report: Reviewer response for version 1

**DOI:** 10.21956/mep.19924.r30209

**Published:** 2019-10-21

**Authors:** Trevor Gibbs

**Affiliations:** 1 AMEE

This review has been migrated. The reviewer awarded 3 stars out of 5 An interesting and topical paper that draws some interesting conclusions, much of which are not surprising but does add to our literature on the topic.I suppose I was somewhat unsure of the authors' approach to the review- what made the choose the slightly unusual Summon engine- was there a particular advantage of using this search engine over the more usual approaches. I was confused somewhat regarding the key words originally used - why history? and also the exclusion of papers not in the English language, particularly when looking at variants from different coutries and cultures.I learned much from the introductory section which provides a useful set of background reading and very useful to those looking at the topic of professionalism.

Reviewer Expertise:

NA

No decision status is available
